# Global trends and frontiers in iNKT cells: a bibliometric and visualized analysis

**DOI:** 10.3389/fimmu.2025.1618254

**Published:** 2025-07-08

**Authors:** Dan Zhang, Jun Lu

**Affiliations:** ^1^ Department of Medical Oncology, Beijing YouAn Hospital, Capital Medical University, Beijing, China; ^2^ Laboratory for Clinical Medicine, Capital Medical University, Beijing, China

**Keywords:** invariant natural killer T cells, bibliometric, antigen, immune response, immunotherapy

## Abstract

**Background:**

Invariant natural killer T (iNKT) cells are an unconventional lymphocyte subset that has garnered increasing attention due to their shared features with both natural killer cells and conventional T cells, as well as their unique dual immunological functions. In this study, we conducted a comprehensive bibliometric analysis to trace the evolution of research in the iNKT cell field, identify emerging trends, and highlight current research hotspots and frontier directions.

**Methods:**

We performed a literature search in the Web of Science Core Collection database to retrieve all publications related to iNKT cells published to December 31, 2024. We then used the visualization tools CiteSpace and VOSviewer to conduct a bibliometric analysis of the retrieved data.

**Results:**

We identified 2,579 relevant publications authored by 12,108 individuals from 2,218 institutions across 70 countries. These publications appeared in 540 journals and collectively cited 60,342 references from 4,322 different journals. The publication volume in the iNKT cell field has significantly increased since 2008, peaking at 151 articles in 2018. This surge highlights the sharp rise of research interest in this area. The United States led in publication output within this field. Among the journals, the Journal of Immunology was the most prolific and also ranked first in total citations. Besra was the most published author, while Bendelac’s research was highly influential. Research on iNKT cells is undergoing a paradigm shift from mechanistic exploration to clinical application.

**Conclusions:**

Our bibliometric analysis delineates the thematic evolution within the iNKT cell research landscape. Future investigations will converge on several pivotal frontiers, including improving the tumor microenvironment, reprogramming the functional activity of iNKT cells within tumors, and advancing engineered immunotherapies. Additionally, strategies to engineer iNKT cells for more targeted and effective therapeutic interventions are likely to gain momentum, as researchers aim to overcome the current limitations in the field and transition from basic mechanistic studies to more impactful clinical applications.

## 1 Introduction

**Figure 1:**
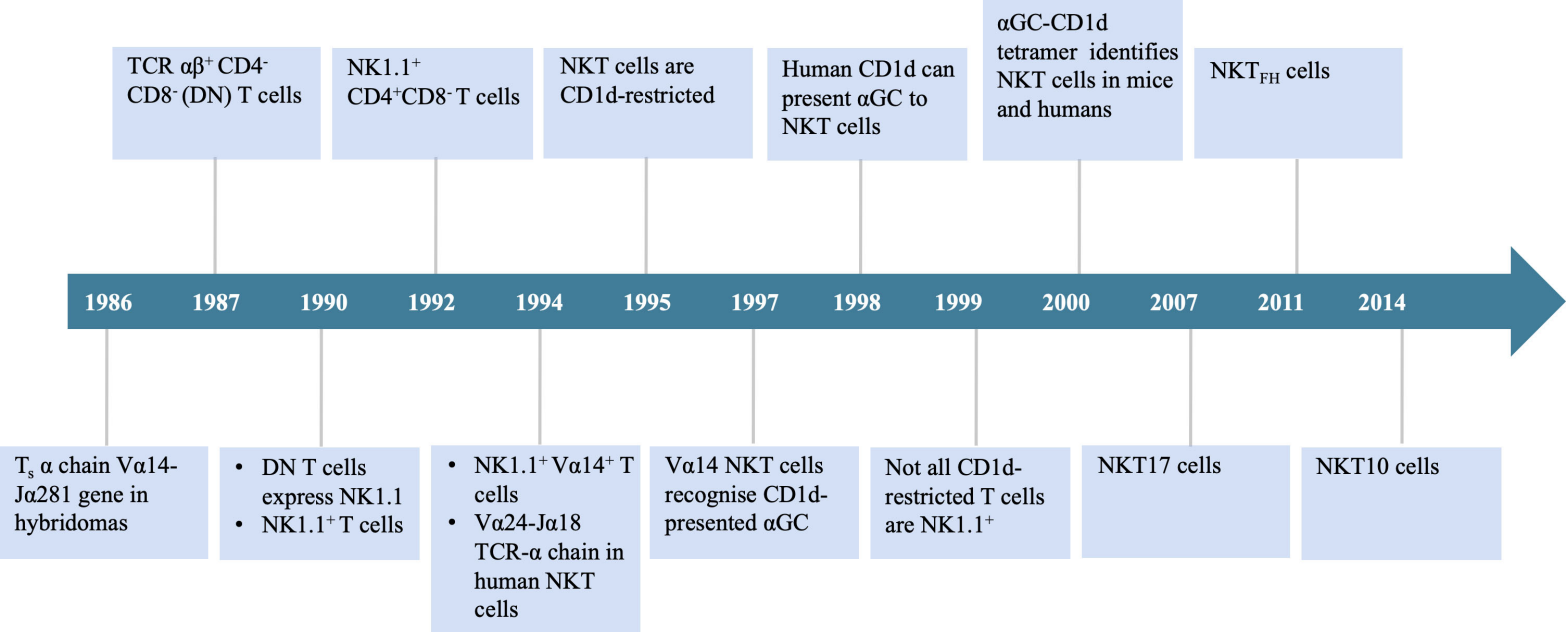
Timeline of important events in the origination of iNKT cells. TCR, T cell receptor; DN, double negative; αGC, α-galactosylceramide.

Invariant natural killer T (iNKT) cells are a specialized subset of lymphocytes characterized by an invariant T cell receptor (TCR) α chain. These cells possess a distinctive recognition system that combines features of both T cells and natural killer (NK) cells, enabling them to bridge innate and adaptive immunity. iNKT cells were first identified in the 1980s, and **Figure 1** presents a timeline of key milestones in their discovery and characterization.

In 1986, Taniguchi et al. (1) identified murine T cells expressing an invariant α chain (later designated Vα14-Jα18 in mice) and cloned the Vα14 gene, which became recognized as a defining feature of this lymphocyte population. In 1987, Fowlkes et al. (2) and Budd et al. (3) independently reported a distinct population of TCRαβ^+^CD4^-^CD8^-^ double-negative (DN) T cells predominantly utilizing the Vβ8 gene segments. By 1990, multiple research groups had demonstrated that this DN T cell population also expressed the NK1.1 surface marker. These findings led to the conclusion that this cell type was distinct from conventional T cells. Researchers subsequently named them “NK1.1 T cells” (4–6).

In 1994, Lantz and Bendelac (7) established a Vβ8.2^+^NK1.1^+^ thymic hybridoma. Their analysis of the TCR repertoire in this cell subset revealed expression of the invariant Vα14 receptor, identical to the Vα14^+^ cells identified in 1986. These findings connected the two previously reported cell populations, now recognized as a single population termed “NK1.1^+^Vα14^+^ T cells.” In the same study, they also identified a corresponding population in humans that expresses the invariant α chain Vα24-Jα18. That year, Dellabona et al. (8) discovered the homologous human population expressing Vα24-Jα18/Vβ11 TCR and generated anti-Vα24/Vβ11 mAbs for its identification. This Vα-Jα pairing is highly conserved across both murine and human evolution. In 1995, Bendelac et al. (9) identified CD1d as the antigen-presenting molecule that specifically interacts with this cell population in mice. CD1d is part of the non-polymorphic major histocompatibility complex (MHC) class I family. By 1997, Kawano et al. (10) identified α-galactosylceramide (αGC) as the canonical lipid ligand presented by CD1d. In 1998, Brossay et al. (11) demonstrated that human Vα24^+^ NKT cells are specifically activated by αGC presented via conserved CD1d molecules. By 2000, the development of αGC-CD1d tetramers and fluorescent tetramers enabled precise tracking of NKT cell immune responses. These tools marked a major breakthrough in the identification of NKT cells in both mice and humans (12, 13).

Studies have shown that iNKT cells recognize αGC presented by CD1d in both mice and humans, demonstrating that CD1d-mediated αGC recognition by NKT cells is highly conserved (11). However, subsequent research revealed that not all CD1d-restricted T cells are NK1.1^+^, leading to the hypothesis of NKT heterogeneity (14). Indeed, in 1992, researchers reported that the NK1.1^+^CD4^+^CD8^-^ T cell subset in the mouse thymus overexpresses the Vβ8 TCR genes (15). At the time, NKT cells were believed to primarily include two subsets: DN and CD4^+^CD8^-^ cells. In 1999, Hammond et al. (16) identified three distinct NKT cell subsets (CD4^+^, DN, and CD8^+^), which were differentially distributed across tissues, highlighting the phenotypic and functional heterogeneity of NKT cells (16). In 2000, Behar and Cardell (17) discovered that NKT cells comprise at least two functionally distinct subsets: NKT1 cells, which produce high levels of interleukin-2 (IL-2) and interferon-gamma (IFN-γ), and NKT2 cells, which secrete large amounts of IL-4 but lower levels of IL-2 and IFN-γ. This classification enabled the categorization of NKT cells based on functional profiles. Since then, additional subsets — including NKT17 (18), NKT_FH_ (19), and NKT10 cells (20) — have also been identified. Upon stimulation with αGC, its analogs, or pro-inflammatory cytokines, iNKT cells rapidly activate and secrete a broad range of T-helper 1 (Th1) and T-helper 2 (Th2) cytokines, including IFN-γ and IL-4 (21, 22). In humans, iNKT cells constitute approximately 0.01%–0.1% of circulating T cells, whereas in mice, they account for roughly 0.2% of total lymphocytes (23). iNKT cells are essential for modulating immune responses in various contexts, including infectious diseases, cancer, and autoimmune disorders. Notably, iNKT cell-based immunotherapy has emerged as a promising strategy in cancer treatment. Given their growing therapeutic relevance, it is crucial to obtain a comprehensive, visual, and in-depth understanding of global research trends and emerging focal points in the iNKT cell field.

Bibliometrics is a method used to quantify and analyze the knowledge structure and dynamic progress of a particular field (24, 25). Despite the increasing attention given to iNKT cells, there has been a lack of relevant bibliometric research conducted on the topic. In this study, we present a comprehensive bibliometric analysis of iNKT cell research, identifying the hotspots and key trends. Research on iNKT cells is undergoing a paradigm shift from mechanistic exploration to clinical application. The study shows the increasing interest in the potential of iNKT cells in cancer immunotherapy and antitumor activity.

## 2 Materials and methods

### 2.1 Literature search and data collection

**Figure 2:**
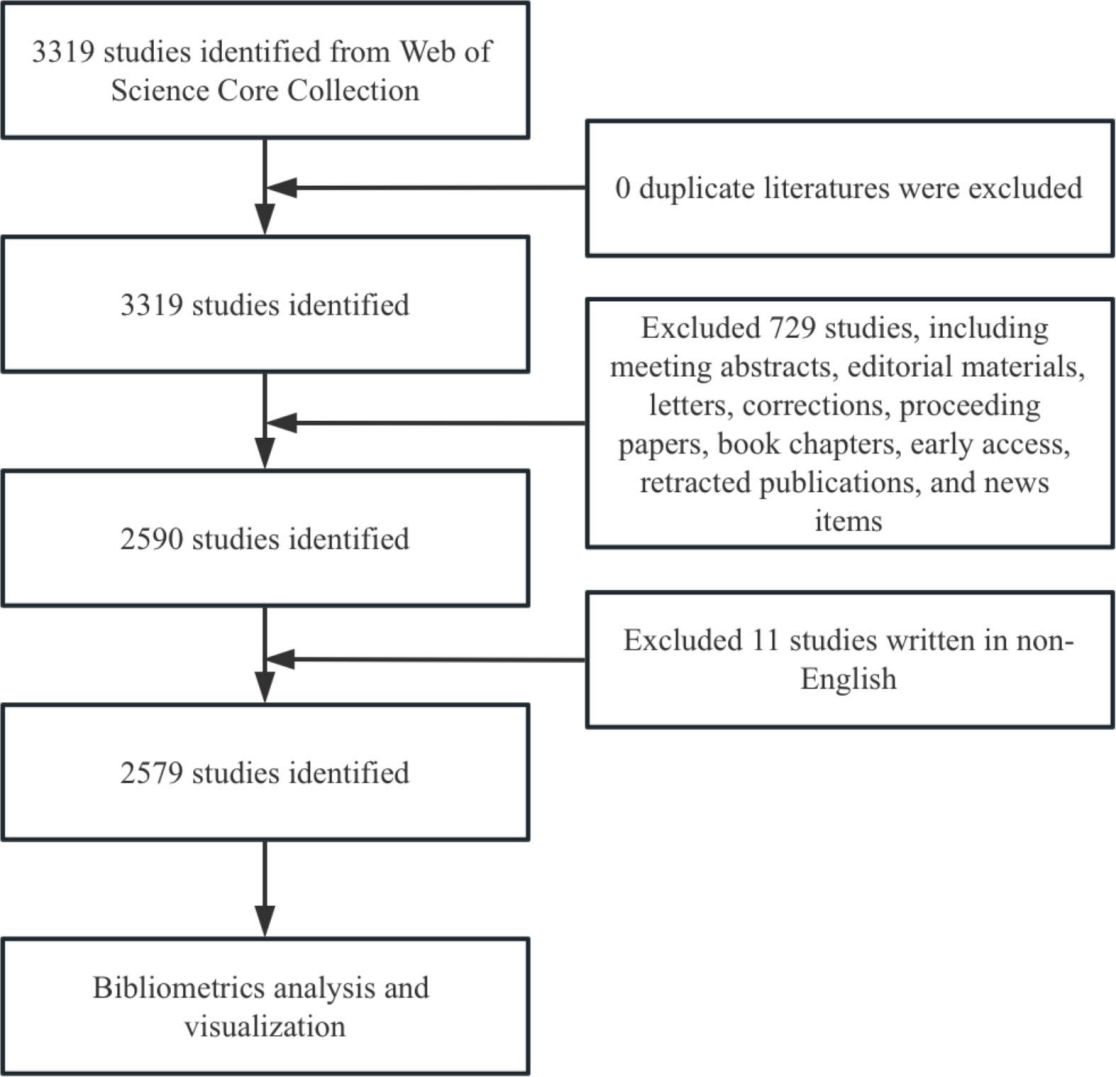
Flowchart of the screening process.

We conducted a literature search in the Web of Science Core Collection (WoSCC) database, covering the period to December 31, 2024. To ensure the inclusion of relevant literature, we used the following search formula: TS = ((“Invariant Natural Killer T-Cells”) OR (“Invariant Natural Killer T Cells”) OR (“Invariant Natural Killer T Cell”) OR (“iNKT Cell”) OR (“Cell, iNKT”) OR (“Cells, iNKT”) OR (“iNKT Cells”) OR (“Valpha14 NKT cells”) OR (“Valpha24 NKT cells”) (“Vα14 NKT cells”) OR (“Vα24 NKT cells”) OR (“CD1d-restricted NKT cells”) OR (“CD1-restricted NKT cells”) OR (“NK1.1 T cells”) OR (“TCRαβ DNT cells”) OR (“Vα14-Jα18”) OR (“Vα24-Jα18”) OR (“Vα24-JαQ”) OR (“Vα14-JαQ”)). The search was limited to original research and review articles, and only English-language publications were included to maintain data quality. After de-duplication, 2,579 documents were retained. The search results were exported in plain text format (**Figure 2**).

### 2.2 Data analysis and visualization

We employed scientific software for data analysis and visualization. To standardize the keyword analysis, we merged synonyms and variations of terms to reduce redundancy. For instance, “iNKT cells” and “invariant NKT cells” were combined. Data tabulation and plotting, based on annual publications and citations, were performed using Excel 2018. Global publication distribution was mapped using R Studio (v2024.09.0). CiteSpace (v6.3.3) was used for visualizing and analyzing institutions, authors, references, and keywords, as well as generating journal dual-map overlays. We performed cluster analysis of countries and institutions using VOSviewer (v1.6.20).

## 3 Results

The 2,579 articles included in this study were authored by 12,108 researchers from 2,218 institutions across 70 countries. These articles were published in 540 journals and cited 60,342 papers from 3,873 journals.

### 3.1 Publication volume and trends

**Figure 3:**
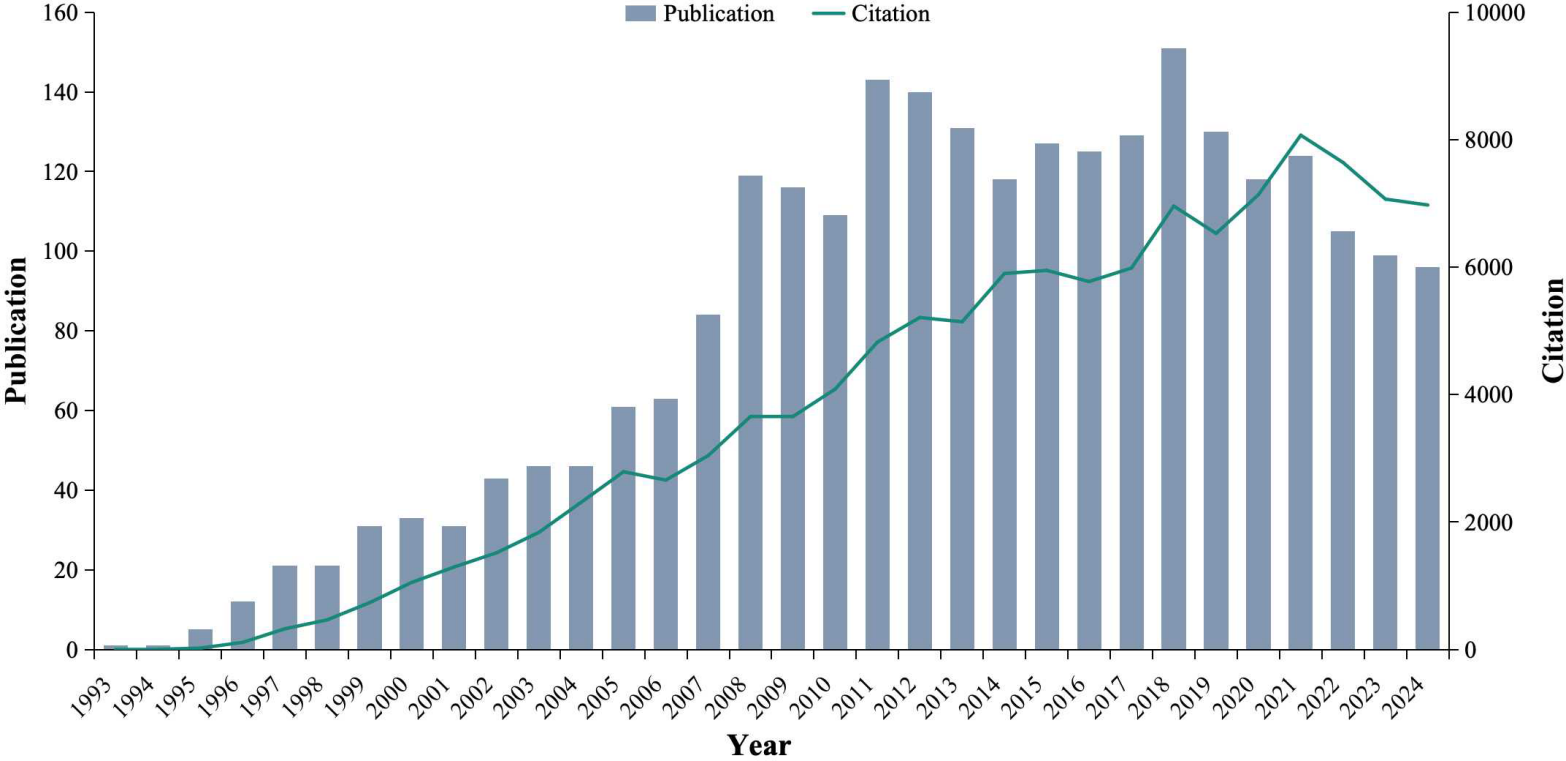
Temporal distribution map of the included publications and citations.

From 1993 to 2024, a total of 2,579 articles related to iNKT cells were retrieved. The number of publications reflects the growing interest and development of this research field. Prior to 1996, iNKT cell research received limited attention, with fewer than 10 publications per year. However, from 2008 onward, the number of publications began to rise significantly, reaching a peak of 151 articles in 2018. Similarly, the number of citations has steadily increased, peaking at 8071 in 2021 (**Figure 3**). These trends suggest that iNKT cell research is a rapidly emerging and evolving field, garnering increasing attention from the scientific community.

### 3.2 Distribution of countries and institutions

**Figure 4:**
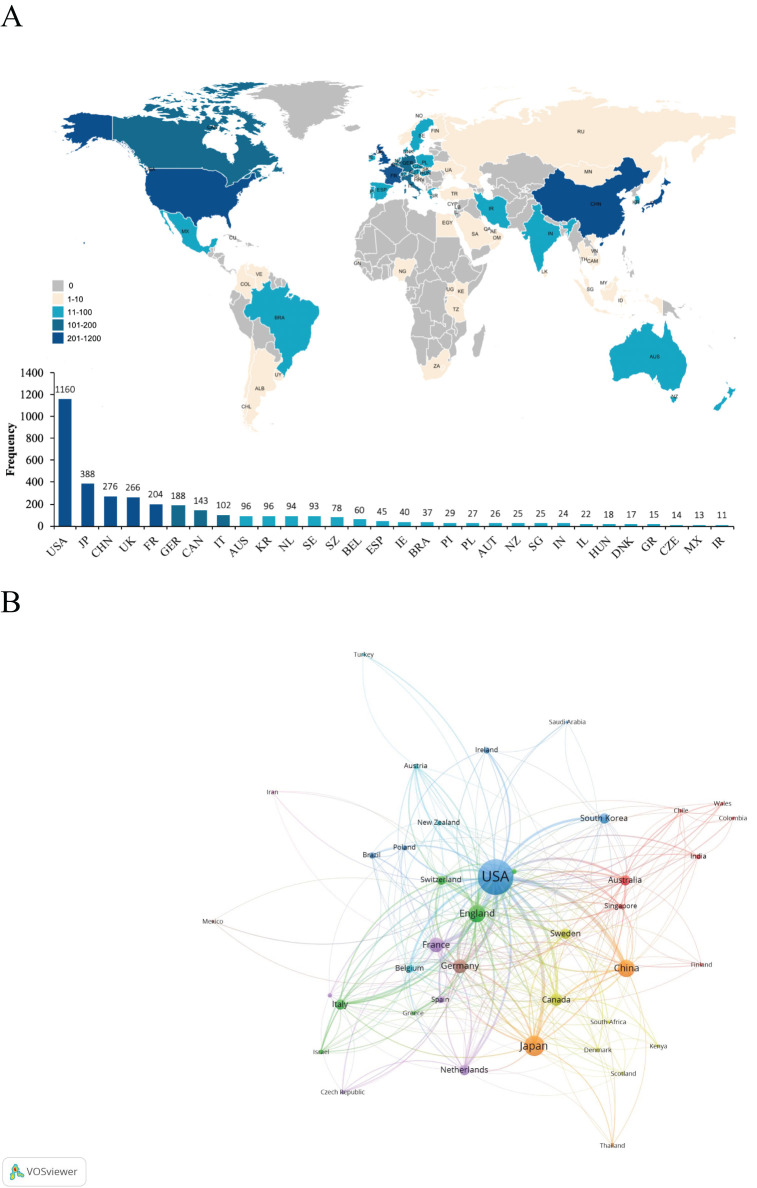
**(A)** Global distribution map of publications. **(B)** Clustered network map of countries.

A total of 70 countries contributed to the research on iNKT cells. **Table 1** presents the top ten countries based on total publication output. The USA was the leading contributor, with 1166 publications, accounting for 45.21% of the total. Japan followed with 388 publications, followed by China (n = 276) and the UK (n = 266). The global distribution map of publications (**Figure 4A**) shows that the countries most active in iNKT cell research are primarily located in North America, Asia, and Europe. The USA also had the highest number of citations (n = 70,418), followed by Japan (n = 18,085) and the UK (n = 14,625).

**Table 1:** Publications in the top 10 countries with the most research output.

Rank	Country	Count (%)	Citation	Top 1% papers	Centrality	Total link strength
1	USA	1160 (44.98%)	70418	20	0.31	807
2	Japan	388 (15.04%)	18085	0	0.11	201
3	China	276 (10.70%)	4847	0	0.02	153
4	UK	266 (10.31%)	14625	1	0.27	343
5	France	204 (7.91%)	12703	0	0.22	178
6	Germany	188 (7.29%)	9088	0	0.26	250
7	Canada	143 (5.54%)	6184	0	0.08	140
8	Italy	102 (3.96%)	3744	0	0.05	79
9	Australia	96 (3.72%)	6831	4	0.13	85
10	South Korea	96 (3.72%)	3283	0	0.02	51

The clustered network map of countries (**Figure 4B**) illustrates the cooperation patterns among nations. In this map, nodes represent individual countries, with the size of each node reflecting the number of publications from that country. The connecting lines between nodes indicate collaborative relationships, with the line thickness representing the strength of these connections. The clusters, represented by different colors, are based on co-citation networks between countries. Stronger collaboration is indicated by thicker lines and greater link strength. The USA collaborates most frequently with the UK (link strength of 108), followed by China (link strength of 81) and Germany (link strength of 73). China primarily collaborates with the USA, followed by Japan (link strength of 15) and the UK (link strength of 11). As shown in **Table 1**, the USA excels in international collaboration, with a total link strength of 807. Centrality measures the importance of a node within a network, indicating its role as a “bridge.” The USA has the highest centrality score, underscoring its pivotal role in international cooperation in this field. While China has a high publication count, its centrality is only 0.02, suggesting that its research is relatively autonomous and independent within the global network.

A total of 2,218 institutions contributed to the publication of 2,579 iNKT cell-related papers. Half of the top ten institutions are from the USA (**Table 2**). Harvard University leads with 133 publications, representing approximately 5.16% of the total output, and has maintained consistent productivity in recent years. The La Jolla Institute for Allergy and Immunology (USA) and the University of Birmingham (UK) ranked second and third, respectively, in terms of publication output.

**Table 2:** Publications in the top 10 institutions with the most research output.

Rank	Institution	Country	Count (%)	Citation	Centrality	Total link strength
1	Harvard University	USA	133 (5.16%)	14446	0.11	126
2	La Jolla Institute for Allergy andImmunology	USA	101 (3.92%)	8270	0.23	129
3	University of Birmingham	UK	99 (3.84%)	5809	0.10	136
4	University of Oxford	UK	72 (2.79%)	4555	0.13	60
5	Chiba University	Japan	67 (2.60%)	5621	0.14	40
6	Vanderbilt University	USA	63 (2.44%)	4548	0.13	57
7	Albert Einstein College of Medicine	USA	58 (2.25%)	2169	0.09	83
8	University of Paris Descartes	France	53 (2.06%)	3629	0.07	79
9	Brigham and Women’s Hospital	USA	52 (2.02%)	4302	0.08	78
10	Institut de la santé et de la recherche médicale	France	47 (1.82%)	2752	0.06	71

**Figure 5:**
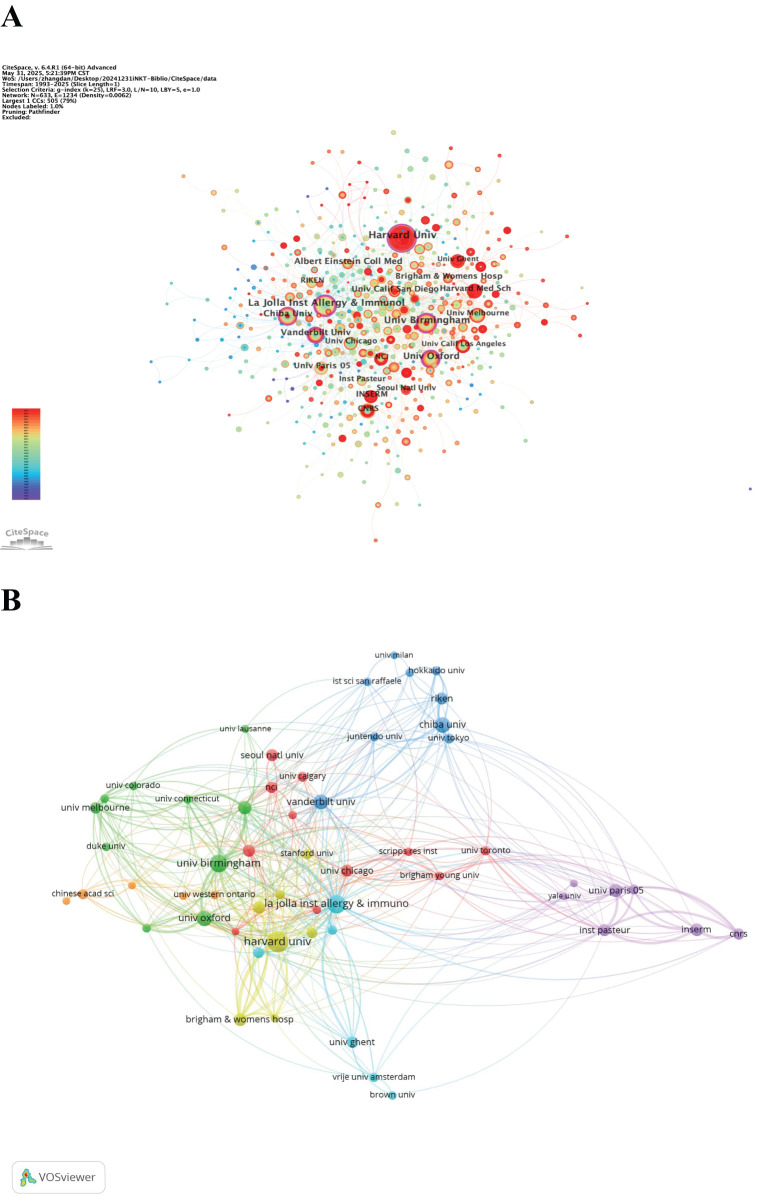
**(A)** Network visualization map of institutional co-occurrence. **(B)** Clustered network map of the institutions.

In the institutional co-occurrence network map (**Figure 5A**), the size of the nodes corresponds to the number of publications, with larger nodes indicating higher output. The red ring highlights institutions that have seen an explosive increase in publications over time. Nodes with a centrality greater than 0.1 are marked with an outer purple ring. The La Jolla Institute for Allergy and Immunology has the highest centrality (0.23) among all institutions. The institutional clustered network map (**Figure 5B**) reveals a strong collaborative network between various institutions. Notably, the University of Birmingham demonstrates the highest tendency to collaborate with other institutions, with a total link strength of 136.

### 3.3 Analysis of journals and co-cited journals

**Figure 6:**
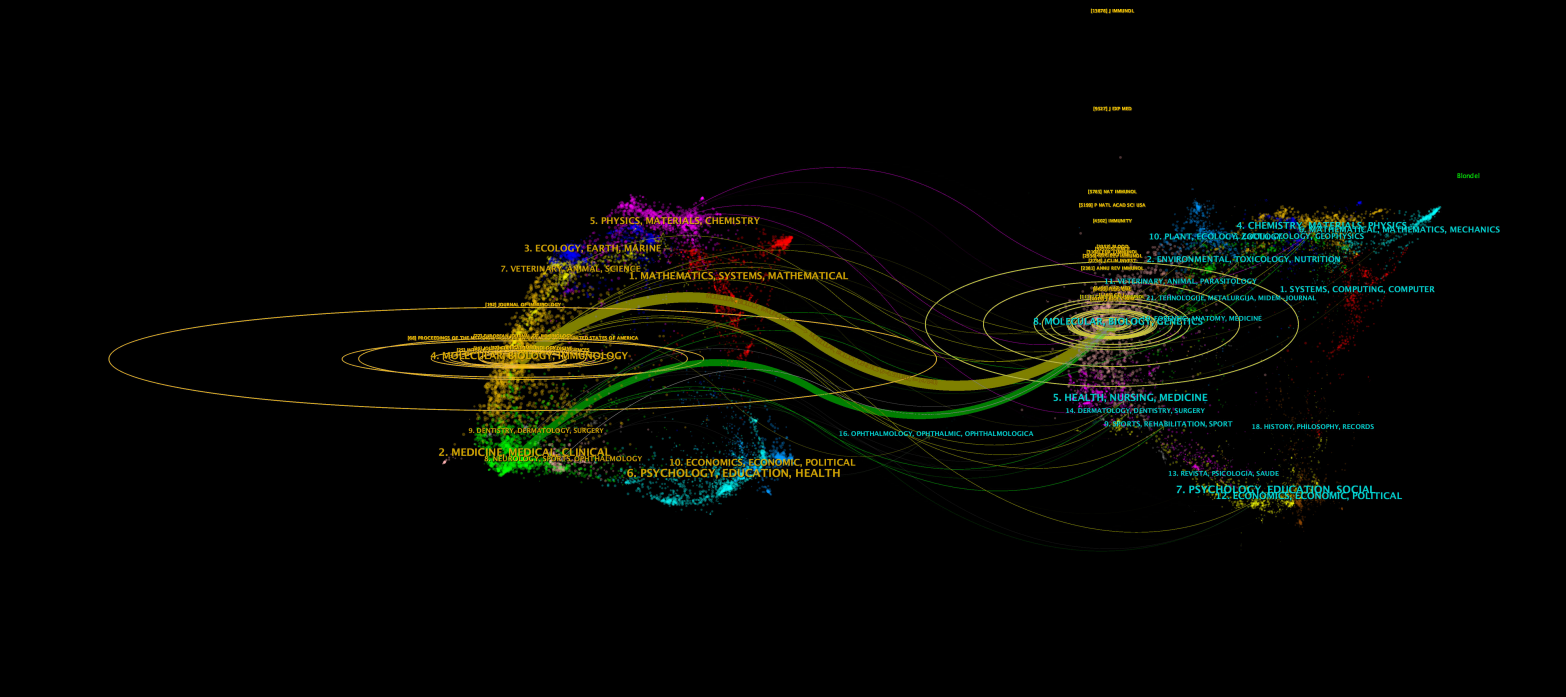
Dual-map overlay of journals.

A total of 2,579 papers on iNKT cells were published across 540 journals. **Table 3** lists the top ten journals by publication volume, with six falling within the JCR Q1 division. The Journal of Immunology led with 298 publications, followed by Frontiers in Immunology and the European Journal of Immunology. Notably, the Journal of Immunology also ranked first in terms of co-citations, receiving 2,422 citations. Six of the top ten co-cited journals had an impact factor greater than 20, with Nature Reviews Immunology being the most influential (IF 67.7). The journal dual-map overlay (**Figure 6**) illustrates the citation relationships between journals. Each journal is represented by a dot, with the left side indicating the citing journal and the right side showing the co-cited journal. The map reveals clusters that correspond to different themes. The yellow and green curves represent citation connections between journals with varying thematic focuses. This figure highlights the evolution of iNKT cell research, transitioning from a focus on “Molecular, Biology, Genetics” to “Molecular, Biology, Immunology” and “Medicine, Medical, Clinical.”

**Table 3:** Top 10 journals and co-cited journals for iNKT cell research.

Rank	Journal	Count (%)	IF (2023)	JCR	Co-cited Journal	Citation	IF (2023)	JCR
1	Journal of Immunology	298 (11.55%)	3.6	Q2	Journal of Immunology	2422	3.6	Q2
2	Frontiers in Immunology	172 (6.67%)	5.7	Q1	Journal of Experimental Medicine	2284	12.6	Q1
3	European Journal of Immunology	107 (4.15%)	4.5	Q2	Proceedings of the National Academy of Sciences of the United States of America	2035	9.4	Q1
4	Proceedings of the National Academy of Sciences of the United States of America	78 (3.02%)	9.4	Q1	Science	1812	44.7	Q1
5	PLoS One	76 (2.95%)	2.9	Q1	Nature Immunology	1749	27.7	Q1
6	Journal of Experimental Medicine	57 (2.21%)	12.6	Q1	European Journal of Immunology	1727	4.5	Q2
7	Blood	46 (1.78%)	21	Q1	Immunity	1682	25.5	Q1
8	Immunology	42 (1.63%)	4.9	Q2	Nature	1652	50.5	Q1
9	Clinical Immunology	39 (1.51%)	4.5	Q2	Annual Review of Immunology	1649	26.9	Q1
10	Nature immunology	34 (1.32%)	27.8	Q1	Nature Reviews Immunology	1570	67.7	Q1

JCR, journal citation reports.

### 3.4 Analysis of authors and co-cited authors

A total of 12,108 authors have contributed to 2,579 publications in the field of iNKT cells. **Table 4** lists the top ten authors by publication volume. Five authors have published more than 30 articles, with Besra from the UK leading with 68 publications. **Table 4** highlight the most co-cited authors in the iNKT cell field. The top ten co-cited authors have accumulated more than 300 citations. Notably, Kronenberg and Van Kaer are not only highly co-cited but also among the most productive authors in the field. Among these, Bendelac from the USA has the highest citation count (1201), underscoring his significant impact. Bendelac has a centrality of 0.15, indicating that the author plays a crucial “bridging” role within the iNKT cell research field.

**Table 4:** Top 10 authors and co-cited authors.

Rank	Author	Count (%)	Centrality	Co-Cited author	Co-citation	Centrality
1	Gurdyal S Besra (UK)	68 (2.64%)	0.04	Albert Bendelac (USA)	1201	0.15
2	Masaru Taniguchi (Japan)	65 (2.52%)	0.08	Dale I Godfrey (Australia)	962	0.01
3	Mitchell Kronenberg (USA)	51 (1.98%)	0.02	Tetsu Kawano (Japan)	791	0.02
4	Steven A Porcelli (USA)	45 (1.74%)	0.04	Jennifer L Matsuda (USA)	626	0.01
5	Vincenzo Cerundolo (UK)	37 (1.43%)	0.02	Mitchell Kronenberg (USA)	525	0.01
6	Luc Van Kaer (USA)	28 (1.09%)	0.02	Manfred Brigl (USA)	500	0.01
7	Michael B Brenner (USA)	27 (1.05%)	0.01	Yuzuru Kinjo (Japan)	421	0.01
8	Dirk Elewaut (Belgium)	26 (1.01%)	0.01	Jenny E Gumperz (USA)	404	0.03
9	Mark A Exley (USA)	25 (0.97%)	0.03	Shin-Ichiro Fujii (Japan)	397	0.01
10	François Trottein (France)	21 (0.81%)	0	Patrick J Brennan (USA)	395	0

### 3.5 Analysis of references

**Figure 7:**
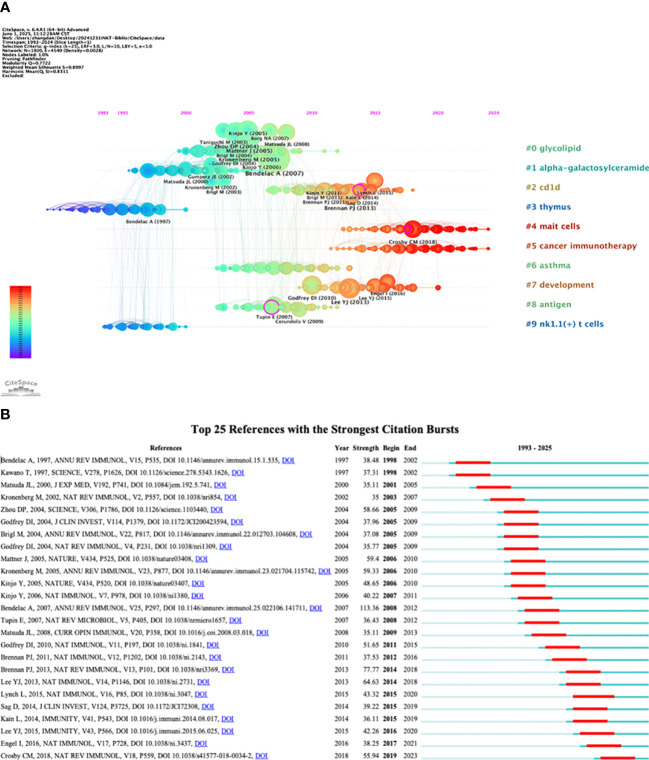
Analysis of co-cited references. **(A)** Timeline view. **(B)** Top 25 references with the strongest citation bursts.

The 2,579 iNKT cell-related papers cited a total of 60,342 references. **Table 5** shows that four of the top five reviews and articles each had more than 100 citations. Among these, the review “The Biology of NKT Cells” published in the Annual Review of Immunology in 2007, and the article “Exogenous and endogenous glycolipid antigens activate NKT cells during microbial infections” published in Nature in 2005, garnered 282 and 151 citations respectively, ranking first among the reviews and articles. A timeline view of reference clustering (**Figure 7A**) organizes the most cited references chronologically, grouping them by thematic clusters. These clusters are ranked by citation count, with smaller numbers indicating larger clusters and greater importance in the field. The duration of each cluster also reflects its persistence over time. **Figure 7A** shows that the top three topics in the references are “#0 glycolipid”, “#1 alpha-galactosylceramide”, and “#2 cd1d”. Around 2005, a large number of studies on glycolipid in iNKT cells were generated. Notably, cluster labeled “#5 cancer immunotherapy” represents a prominent area of research in recent years. The reference burst graph (**Figure 7B**) shows the burst periods, where “Begin” indicates the start of the burst and “Strength” reflects its intensity. It highlights the influence of the 2007 review “The Biology of NKT Cells”, which shows the strongest burst value of 113.36. This suggests that the paper had a significant and lasting impact at the time of publication.

**Table 5:** Top 5 co-cited reviews and articles on iNKT cell research.

Type	Rank	Reference	Year	Journal	Co-Citation	Centrality
Review	1	The biology of NKT cells	2007	Annual Review of Immunology	282	0.01
	2	Invariant natural killer T cells: an innate activation scheme linked to diverse effector functions	2013	Nature Reviews Immunology	158	0.01
	3	Toward an understanding of NKT cell biology: progress and paradoxes	2005	Annual Review of Immunology	146	0.01
	4	Raising the NKT cell family	2010	Nature Immunology	113	0.05
	5	Tissue-specific functions of invariant natural killer T cells	2018	Nature Reviews Immunology	97	0.01
Article	1	Exogenous and endogenous glycolipid antigens activate NKT cells during microbial infections	2005	Nature	151	0.08
	2	Lysosomal glycosphingolipid recognition by NKT cells	2004	Science	134	0.04
	3	Steady-state production of IL-4 modulates immunity in mouse strains and is determined by lineage diversity of iNKT cells	2013	Nature Immunology	127	0.04
	4	Recognition of bacterial glycosphingolipids by natural killer T cells	2005	Nature	123	0
	5	Natural killer T cells recognize diacylglycerol antigens from pathogenic bacteria	2006	Nature Immunology	98	0.01

### 3.6 Analysis of keywords

**Figure 8:**
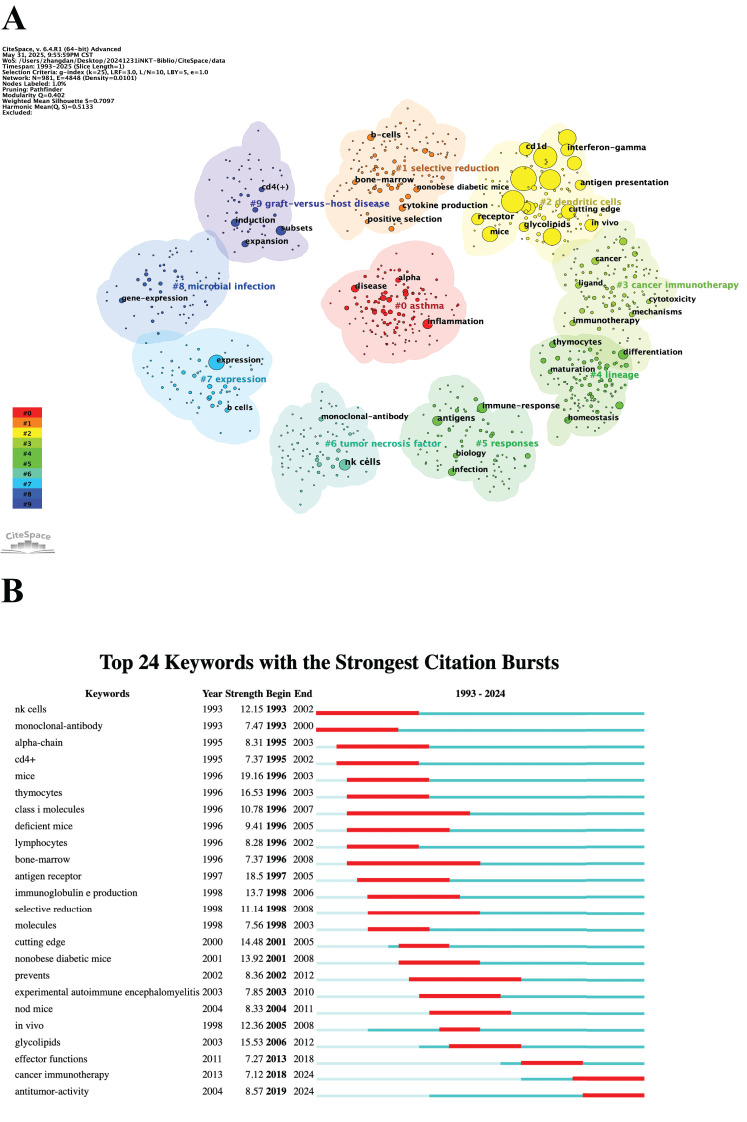
Analysis of keywords. **(A)** Clustered network map. **(B)** Top 24 keywords with the strongest citation bursts.

Keywords reflect the core themes and emerging research hotspots within a field. A total of 981 keywords were extracted from 2,579 documents. **Table 6** lists the top 20 keywords. The most frequently mentioned keywords included “T cells,” “NKT cells,” “iNKT cells,” “activation,” “immunity,” and “dendritic cells (DCs).” We then performed clustering analysis on all the keywords, resulting in ten thematic clusters: “#0 asthma,” “#1 selective reduction,” “#2 DCs,” “#3 cancer immunotherapy,” “#4 lineage,” “#5 responses,” “#6 tumor necrosis factor,” “#7 expression,” “#8 microbial infection,” and “#9 graft-versus-host disease” (**Figure 8A**). Finally, we assessed the burst strength of iNKT cell research across different areas to identify periods of increased research activity (**Figure 8B**). “NK cells” and “monoclonal-antibody” emerged as the earliest burst keywords in 1993, implying that research on NK cells and monoclonal antibodies sparked widespread interest at that time. Between 1996 and 2003, “mice” exhibited the strongest burst with a strength of 19.16, underscoring its significance during that period. A summary of burst keywords up to 2024 reveals that cancer immunotherapy and antitumor activity are emerging as key research directions and hotspots.

**Table 6:** Top 20 keywords on iNKT cell research.

Rank	Keyword	Count	Centrality	Rank	Keyword	Count	Centrality
1	t cells	1089	0.02	11	cd1d	312	0.04
2	nkt cells	939	0.01	12	recognition	290	0.02
3	inkt cells	927	0.02	13	*in vivo*	263	0.04
4	activation	751	0.02	14	interferon-gamma	256	0.06
5	immunity	538	0.01	15	receptor	227	0.06
6	dendritic cells	535	0.01	16	nk cells	223	0.10
7	expression	418	0.03	17	lymphocytes	199	0.10
8	mice	412	0.03	18	innate	174	0.02
9	cutting edge	370	0.03	19	antigen presentation	170	0.06
10	alpha-galactosylceramide	360	0.02	20	antigens	167	0.07

## 4 Discussion

### 4.1 Seminal studies

#### 4.1.1 Contribution of highly co-cited authors

The citation level of an author reflects their influence and contribution to the academic field. Highly co-cited authors’ research has had a profound impact on the development and innovation within the discipline. The most co-cited author in the iNKT cell field is Bendelac from the USA. He has made significant contributions to the discovery and identification of bispecific receptors and lipid ligands, as well as to the developmental pathways and functional regulatory mechanisms in iNKT cells (26–28). His work has been instrumental in guiding and shaping subsequent research. The second most co-cited author is Godfrey from Australia. His team demonstrated that iNKT cells mediate the anti-tumor metastatic activity of αGC via IFN-γ (29). His pioneering research on the development, regulation, and therapeutic potential of unconventional T cells, including Mucosal Associated Invariant (MAIT) cells and iNKT cells, has laid a strong foundation for future clinical applications (30–33). The third most co-cited author is Kawano from Japan. His research team was the first to propose that αGC-mediated stimulation of iNKT cells is restricted by CD1-d and depends on TCR and costimulatory molecules (10). This discovery quickly spawned an abundance of experimental studies exploring the mechanisms of iNKT cell activation, and moreover became the starting point for immunotherapy development.

#### 4.1.2 Impact of highly co-cited references

The number of citations a reference receives reflects its academic significance and influence. A highly co-cited article is often considered a seminal work in its field, with many subsequent studies building upon it. The most frequently co-cited article in this study is “The Biology of NKT Cells,” which provides a comprehensive overview of the biological characteristics of NKT cells, including their types, developmental processes, and functions (34). Published in Annual Review of Immunology in 2007, this review has become a cornerstone reference in the study of iNKT cells. The second most co-cited article is “Invariant Natural Killer T Cells: An Innate Activation Scheme Linked to Diverse Effector Functions,” published in Nature Reviews Immunology in 2013 (35). In this review, the author examines the mechanisms behind iNKT cell activation, focusing on the regulatory roles of lipid antigens, the inflammatory microenvironment, and the interactions between iNKT cells and other immune cells. This work has been foundational in advancing our understanding of iNKT cell functions in both physiological and pathological contexts. The third article is “Exogenous and Endogenous Glycolipid Antigens Activate NKT Cells During Microbial Infections,” published in Nature in 2005 (28). This study identified two new antigenic targets for NKT cells in antibacterial defense: glycosylceramides from the cell wall of *Sphingomonas* and the endogenous lysosomal glycosphingolipid isoglobotrihexosylceramide (iGb3). It also demonstrated that glycosylceramides could serve as an alternative to lipopolysaccharide (LPS) in activating the innate immune system, providing insight into the recognition of Gram-negative and LPS-negative bacterial cell walls. This discovery became a landmark finding in iNKT cell antigen research.

The highly co-cited authors and references analyzed in this study are pivotal to the ongoing research and clinical applications of iNKT cells, such as in vaccine development, autoimmune disease treatments, and cancer immunotherapy.

### 4.2 Research hotspots

Bibliometric analysis can reveal key themes, trends, and gaps in the research field by examining high-frequency keywords in references. In this study, the most frequent keywords primarily relate to three central aspects of iNKT cell research.

#### 4.2.1 Mechanisms of cell activation and antigen presentation

Keywords associated with antigens include “activation,” “DCs,” “expression,” “alpha-galactosylceramide,” “CD1d,” “recognition,” “receptor,” “antigen presentation,” and “antigens.” These terms reflect the early stages of iNKT cell research, when researchers first identified antigens that sparked further investigation into these cells. The study revealed that iNKT cells recognize both exogenous and endogenous lipid antigens. Exogenous antigens include αGC and analogues from environmental sources (36–38), and glycosphingolipids (GSLs) and diacylglycerol lipids from the microbiota, such as those found in *Sphingomonas* (28), *Streptococcus pneumoniae*, Group B *Streptococcus*, *Borrelia burgdorferi* (39), and *Bacteroides fragilis* (40). Endogenous antigens identified to date include β-GalCers, glucosylceramides (GlcCers), phospholipid antigens (such as phosphatidylinositol, phosphatidylethanolamine, and lysophospholipids), iGb3, the gangliosides GM3 and GD3, and peroxisome-derived ether-linked lipids (41–44). However, endogenous antigens activate iNKT cells less efficiently than exogenous antigens like αGC (41).

CD1d is a crucial molecule in the recognition of glycolipid antigens by the TCR of iNKT cells (36). The TCR of iNKT cells consists of α- and β-chains, with the highly conserved invariant α-chain enabling iNKT cells to recognize a broad range of lipid antigen-CD1d complexes. The chemical structure of lipid antigens—such as the type of glycosidic bond, the sugar group, and the length of the lipid acyl chain—affects the affinity and specificity of TCR binding (45). The affinity of the human Vα24/Vβ11 TCR for the αGC+CD1d complex is comparable to that of the classical TCR-MHC interaction, while the affinity of the mouse Vα14/Vβ8 TCR-αGC+CD1d interaction is much higher (46). Studies have shown that most lipids associated with CD1d are non-stimulatory, with sphingomyelins being the predominant type (47). CD1d preferentially binds long-chain sphingolipids and glycerophospholipids, which are non-activating for iNKT cells (48). iNKT cells recognize lipid antigens in a highly versatile manner, allowing them to respond effectively to a wide variety of pathogens. Antigen recognition is fundamental to understanding and applying iNKT cell biology. This study highlights a gap in the research: few studies have defined the exact structure of each antigen or the mechanisms that influence iNKT cell immune responses. In particular, research on endogenous lipids remains scarce and requires urgent attention.

#### 4.2.2 Immune effector functions and transcellular interactions

The primary keywords related to immune response include “T cells,” “NKT cells,” “iNKT cells,” “immunity,” “DCs,” “interferon-gamma,” “NK cells,” and “lymphocytes.” iNKT cells possess both T- and NK-cell-specific recognition mechanisms, enabling them to produce both innate and adaptive immune responses in response to antigenic threats. The innate immune system serves as the body’s first line of defense. iNKT cells are activated through endogenous antigen/CD1d-mediated TCR stimulation and indirect cytokine stimulation (*e.g*., IL-12, IL-18) from antigen-presenting cells activated by pattern recognition receptors (49). Upon activation, iNKT cells rapidly secrete a broad array of cytokines, depending on the tissue environment, including Th1 (IFN-γ, TNF-α), Th2 (IL-4, IL-10, IL-5, IL-13), and Th17 (IL-17A) cytokines. Th1 cytokines drive inflammation and cellular immune responses, Th2 cytokines are linked to humoral immunity and the regulation of autoimmune diseases, and Th17 cytokines enhance host defense against extracellular pathogens (50). By secreting cytokines, iNKT cells quickly initiate inflammatory responses and stimulate innate immune cells, which, in turn, exert various immune effects, including immunomodulation and cytotoxicity (51). iNKT-cell-derived IFN-γ plays a critical role in neutrophil recruitment and bacterial clearance (52). Activated iNKT cells promote DC maturation and polarization through CD40/CD40L interactions (53). IL-12 secreted by DCs enhances both the trans-activation of NK cells by iNKT cells and the secretion of IFN-γ by iNKT cells, which, in turn, stimulates DCs to secrete more IL-12, further amplifying the immune response. Carnaud et al. (54) first demonstrated that NKT cells induce rapid NK cell activation and proliferation through IFN-γ, and this NK cell activation further promotes IFN-γ secretion and enhances cytotoxicity. Thus, iNKT cell activation strengthens the innate immune response, facilitating early control of infections (52).

In addition to their role in innate immunity, iNKT cells are crucial for adaptive immunity by regulating antigen presentation and supporting lymphocyte activation. iNKT cells can directly activate CD4^+^ T cells or do so via DCs, which differentiate into T_FH_ cells that drive B cells to generate early antibody responses (55). iNKT_FH_ cells also provide T_FH_-like help to B cells (56). Furthermore, iNKT cells enhance the maturation of DCs, promoting the cross-presentation of exogenous antigens to CD8^+^ T cells. Activation of iNKT cells also induces strong antitumor effects in CD8^+^ T cells (57, 58) and helps prevent malaria by inducing liver-resident memory CD8^+^ T cells (59).

#### 4.2.3 Disease modeling and preclinical research

The high frequency keywords “mice,” “*in vivo*,” “expression,” and “cutting edge” reflect the importance of animal models and gene expression research. Gene-knockout animal models (e.g., CD1d^-^/^-^ and Jα18^-^/^-^ mice) underpin fundamental research into iNKT cell biology. By genetically ablating the development or functional capacity of iNKT cells, these models have propelled transformative advances spanning from mechanistic dissection of immune pathways to clinical translation of targeted therapies.

At the functional mechanism level, gene-knockout mouse models serve as the gold standard for investigating the biological functions of iNKT cells. In a seminal study, Kawano et al. (10) identified αGC as a specific TCR activator of iNKT cells through comparative analysis of splenocytes from wild-type mice, Vα14 NKT-deficient mice, Vα14 NKT mice, and NK cell-only mice. The CD1d^-^/^-^ mouse model (lacks all CD1d-dependent NKT cells) has been instrumental in establishing iNKT cells as a critical bridge between innate and adaptive immunity. Using CD1d-deficient mice as negative controls, researchers confirmed that αGC activates NKT cells via CD1d-restricted mechanisms, thereby inducing potent NK cell activity and robust cytokine production (54). Subpopulation-specific defect models (e.g., conditional knockouts of *T-bet* for iNKT1, *GATA3* for iNKT2, or *PLZF* for iNKT10) have significantly advanced our understanding of functional heterogeneity among iNKT cell subsets (60–62). Methodologically, these animal models have provided essential validation tools—for instance, CD1d^-^/^-^ mice served as negative controls to confirm the specificity of CD1d tetramer staining, enabling high-precision isolation of iNKT cells (12, 13).

iNKT cell-deficient mouse models serve as indispensable tools in disease therapeutics, providing in-depth mechanistic insights into the bidirectional immunoregulatory roles of iNKT cells across metabolic disorders, autoimmune diseases, infections, and cancers (63–66). These models establish critical preclinical platforms for developing targeted therapeutic strategies. Besides, humanized mouse models are widely employed in iNKT cell-based therapy research (67, 68). Translational advancements—ranging from αGC dosing optimization to CAR-iNKT cell design—heavily rely on validation through genetically deficient animal models. Preclinical data derived from murine systems demonstrate that allogeneic CAR-iNKT cells not only exert direct cytotoxicity but also prime host CD8^+^ T cell-mediated antitumor responses, thereby amplifying therapeutic efficacy (69). This dual-action mechanism provides a robust safety foundation for human clinical trials. Collectively, these studies elucidate the dynamic functional evolution of iNKT cells during disease pathogenesis and establish a conceptual framework for clinical translation.

### 4.3 Emerging research trends

Research on iNKT cells has become a prominent focus in immunology, with growing interest in understanding their underlying mechanisms. Initial studies primarily centered on the discovery and characterization of iNKT cells. This was followed by investigations into their immune functions, and more recently, a surge in exploring their therapeutic potential. Keyword burst analysis has highlighted the current frontiers of iNKT cell research, shedding light on the key issues currently driving academic interest.

#### 4.3.1 Cancer immunotherapy

Owing to their potent antitumor activity and favorable safety profile, iNKT cell-based therapies have emerged as promising modalities in cancer immunotherapy. Activated iNKT cells can directly kill CD1d^+^ tumor cells by releasing perforin and granzyme B, as well as through Fas/FasL interactions. Additionally, iNKT cells can promote tumor cell lysis indirectly by recruiting NK cells, DCs, and cytotoxic T lymphocytes (70). These dual mechanisms underpin their therapeutic potential across diverse malignancies, such as hepatocellular carcinoma (71), T-cell lymphoma (72), neuroblastoma (73), and melanoma (74).

To harness this potential, multiple strategies are advanced. Initially, autologous infusion of ex vivo activated iNKT cells replenishes dysfunctional populations, restoring antitumor immunity (71). For instance, a Phase I clinical trial in stage IIIB-IV melanoma patients demonstrated that adoptive iNKT transfer elicited Th1-polarized responses with tumor regression (74). Subsequently, CAR-iNKT engineering was developed to enhance specificity (75, 76). Critically, the intrinsic ability of iNKT cells to recognize CD1d-presented endogenous tumor lipids via their invariant TCR creates a dual-targeting synergy: this not only amplifies CAR-mediated cytotoxicity but also mitigates antigen escape by engaging lipid-specific killing pathways (77). It’s reported that anti-TCRVβ CAR-iNKT cells exhibited enhanced tumor clearance and reduced tumor escape in T cell lymphoma models compared to conventional CAR-T cells (72). More recently, novel activation platforms overcome endogenous limitations. The bispecific nanobody VHH1D12 bridges CD1d and invariant TCR to stabilize this interaction, enhancing endogenous lipid antigen presentation without requiring exogenous αGC. Its unique advantages include selective iNKT activation and suppression of type II NKT cells, with preclinical efficacy surpassing traditional agonists (78).

Beyond direct cell therapies, iNKT cells function as potent molecular adjuvants for cancer vaccines (79). These cells can help augment CD8^+^ T cell responses to co-presented peptides, much like conventional CD4^+^ T cells. *In vitro*, administering DCs loaded with αGC promotes long-term IFN-γ production and prevents iNKT cell dysfunction, leading to a more robust antitumor effect (51). Common vaccine types primarily include lipid-based antigenic vaccines that activate iNKT cell activity, such as αGC and its analogs (80). These CD1d-binding vaccines activate iNKT cells, which then exert adjuvant effects to enhance the immunogenicity against bacterial (81, 82), viral (83, 84), parasitic (85), and tumor (80, 86, 87) antigens.

#### 4.3.2 Antitumor activity

As previously described, iNKT cells exert antitumor activity through dual mechanisms of direct cytotoxicity and immune microenvironment remodeling. Leveraging their inherent capacity for dual-targeting synergy—simultaneous engagement of protein antigens via CAR and CD1d-presented lipid antigens via their invariant TCR—coupled with robust tumor microenvironment (TME) remodeling, iNKT cells effectively overcome barriers in solid tumor therapy.

Nevertheless, TME-imposed immunosuppression and metabolic dysfunction critically constrain their efficacy. Prevalent inhibitory signals, including PD-L1 checkpoint activation and adenosine-driven A_2_AR signaling, impair iNKT cell function, necessitating innovative countermeasures (88, 89). Notably, Liu et al. (90) demonstrated that IL-15 co-expression in iNKT cells confers resistance to tumor-associated macrophage-mediated immunosuppression, significantly enhancing anti-metastatic efficacy—a strategy now informing next-generation CAR-iNKT designs. Concurrently, TME-driven metabolic stress induces iNKT cell dysfunction, where metabolic reprogramming disrupts signaling pathways and impairs intratumoral motility and activation (91, 92). This dysfunction is counteracted by PPARγ activation via pioglitazone, which rescues iNKT function through restoration of lipid biosynthesis (e.g., cholesterol) (93).

Furthermore, while many malignancies downregulate CD1d to evade immune recognition, emerging strategies enable potent circumvention. Studies in advanced B-cell malignancies show that a soluble CD1d-CD19 fusion protein activates iNKT cells independently of endogenous CD1d, augmenting antitumor efficacy (94). Recent work in aggressive cholangiocarcinoma further revealed that Vorinostat (histone deacetylase inhibitor) restores iNKT function by reversing epigenetic silencing of CD1d and upregulating its expression (95).

Collectively, these advances transform iNKT cells into multifunctional therapeutic agents capable of overcoming the TME’s most recalcitrant immunosuppressive and metabolic barriers.

## 5 Limitations

This study has several limitations related to its bibliometric approach. All data were sourced from the WoSCC database. While this database is comprehensive and up to date, it may not include all relevant publications, potentially introducing a selectivity bias. Additionally, some literature included in this study may not have accumulated sufficient citations due to its recent publication, which could lead to an underestimation of its impact. Moreover, there may be a time lag in the data, but the results remain reliable and can offer valuable insights for guiding future research directions.

## 6 Conclusion

Our analysis reveals a paradigm shift in iNKT cell research—from mechanistic exploration to clinical implementation. Studies conducted by American researchers continue to dominate the field, with the Journal of Immunology publishing the wealthiest research in the field. Bendelac was a highly influential researcher in this area. These data-driven insights provide an evidence-based roadmap for accelerating iNKT cell translation, directing resources toward optimizing therapies for solid tumors and advancing engineering strategies. These efforts aim to overcome the challenges posed by the TME, improve the persistence and function of iNKT cells within tumors, and ultimately achieve more effective and targeted therapeutic interventions.

## Data Availability

The raw data supporting the conclusions of this article will be made available by the authors, without undue reservation.

## References

[1] Imai K Kanno M Kimoto H Shigemoto K Yamamoto S Taniguchi M. Sequence and expression of transcripts of the T-cell antigen receptor alpha-chain gene in a functional, antigen-specific suppressor-T-cell hybridoma. Proc Natl Acad Sci U S A. (1986) 83:8708–12. doi: 10.1073/pnas.83.22.8708, PMID: 2946043 PMC387000

[2] Fowlkes BJ Kruisbeek AM Ton-That H Weston MA Coligan JE Schwartz RH. A novel population of T-cell receptor alpha beta-bearing thymocytes which predominantly expresses a single V beta gene family. Nature. (1987) 329:251–4. doi: 10.1038/329251a0, PMID: 3114646

[3] Budd RC Miescher GC Howe RC Lees RK Bron C MacDonald HR. Developmentally regulated expression of T cell receptor beta chain variable domains in immature thymocytes. J Exp Med. (1987) 166:577–82. doi: 10.1084/jem.166.2.577, PMID: 3496420 PMC2189605

[4] Sykes M. Unusual T cell populations in adult murine bone marrow. Prevalence of CD3+CD4-CD8- and alpha beta TCR+NK1.1+ cells. J Immunol. (1990) 145:3209–15. doi: 10.4049/jimmunol.145.10.3209, PMID: 1977798

[5] Ballas ZK Rasmussen W. NK1.1+ thymocytes. Adult murine CD4-, CD8- thymocytes contain an NK1.1+, CD3+, CD5hi, CD44hi, TCR-V beta 8+ subset. J Immunol. (1990) 145:1039–45. doi: 10.4049/jimmunol.145.4.1039, PMID: 1696293

[6] Levitsky HI Golumbek PT Pardoll DM. The fate of CD4-8- T cell receptor-alpha beta+ thymocytes. J Immunol. (1991) 146:1113–7. doi: 10.4049/jimmunol.146.4.1113, PMID: 1825103

[7] Lantz O Bendelac A. An invariant T cell receptor alpha chain is used by a unique subset of major histocompatibility complex class I-specific CD4+ and CD4-8- T cells in mice and humans. J Exp Med. (1994) 180:1097–106. doi: 10.1084/jem.180.3.1097, PMID: 7520467 PMC2191643

[8] Dellabona P Padovan E Casorati G Brockhaus M Lanzavecchia A. An invariant V alpha 24-J alpha Q/V beta 11 T cell receptor is expressed in all individuals by clonally expanded CD4-8- T cells. J Exp Med. (1994) 180:1171–6. doi: 10.1084/jem.180.3.1171, PMID: 8064234 PMC2191638

[9] Bendelac A Lantz O Quimby ME Yewdell JW Bennink JR Brutkiewicz RR. CD1 recognition by mouse NK1+ T lymphocytes. Science. (1995) 268:863–5. doi: 10.1126/science.7538697, PMID: 7538697

[10] Kawano T Cui J Koezuka Y Toura I Kaneko Y Motoki K. CD1d-restricted and TCR-mediated activation of valpha14 NKT cells by glycosylceramides. Science. (1997) 278:1626–9. doi: 10.1126/science.278.5343.1626, PMID: 9374463

[11] Brossay L Chioda M Burdin N Koezuka Y Casorati G Dellabona P. CD1d-mediated recognition of an alpha-galactosylceramide by natural killer T cells is highly conserved through mammalian evolution. J Exp Med. (1998) 188:1521–8. doi: 10.1084/jem.188.8.1521, PMID: 9782129 PMC2213408

[12] Matsuda JL Naidenko OV Gapin L Nakayama T Taniguchi M Wang CR. Tracking the response of natural killer T cells to a glycolipid antigen using CD1d tetramers. J Exp Med. (2000) 192:741–54. doi: 10.1084/jem.192.5.741, PMID: 10974039 PMC2193268

[13] Benlagha K Weiss A Beavis A Teyton L Bendelac A. *In vivo* identification of glycolipid antigen-specific T cells using fluorescent CD1d tetramers. J Exp Med. (2000) 191:1895–903. doi: 10.1084/jem.191.11.1895, PMID: 10839805 PMC2213523

[14] Chiu YH Jayawardena J Weiss A Lee D Park SH Dautry-Varsat A. Distinct subsets of CD1d-restricted T cells recognize self-antigens loaded in different cellular compartments. J Exp Med. (1999) 189:103–10. doi: 10.1084/jem.189.1.103, PMID: 9874567 PMC1887692

[15] Hayakawa K Lin BT Hardy RR. Murine thymic CD4+ T cell subsets: a subset (Thy0) that secretes diverse cytokines and overexpresses the V beta 8 T cell receptor gene family. J Exp Med. (1992) 176:269–74. doi: 10.1084/jem.176.1.269, PMID: 1351921 PMC2119274

[16] Hammond KJ Pelikan SB Crowe NY Randle-Barrett E Nakayama T Taniguchi M. NKT cells are phenotypically and functionally diverse. Eur J Immunol. (1999) 29:3768–81. doi: 10.1002/(SICI)1521-4141(199911)29:11<3768::AID-IMMU3768>3.0.CO;2-G, PMID: 10556834

[17] Behar SM Cardell S. Diverse CD1d-restricted T cells: diverse phenotypes, and diverse functions. Semin Immunol. (2000) 12:551–60. doi: 10.1006/smim.2000.0273, PMID: 11145861

[18] Michel ML Keller AC Paget C Fujio M Trottein F Savage PB. Identification of an IL-17-producing NK1.1(neg) iNKT cell population involved in airway neutrophilia. J Exp Med. (2007) 204:995–1001. doi: 10.1084/jem.20061551, PMID: 17470641 PMC2118594

[19] Chang PP Barral P Fitch J Pratama A Ma CS Kallies A. Identification of Bcl-6-dependent follicular helper NKT cells that provide cognate help for B cell responses. Nat Immunol. (2011) 13:35–43. doi: 10.1038/ni.2166, PMID: 22120117

[20] Sag D Krause P Hedrick CC Kronenberg M Wingender G. IL-10-producing NKT10 cells are a distinct regulatory invariant NKT cell subset. J Clin Invest. (2014) 124:3725–40. doi: 10.1172/JCI72308, PMID: 25061873 PMC4151203

[21] Qin Y Oh S Lim S Shin JH Yoon MS Park SH. Invariant NKT cells facilitate cytotoxic T-cell activation via direct recognition of CD1d on T cells. Exp Mol Med. (2019) 51:1–9. doi: 10.1038/s12276-019-0329-9, PMID: 31653827 PMC6814837

[22] Cui G Shimba A Jin J Ogawa T Muramoto Y Miyachi H. A circulating subset of iNKT cells mediates antitumor and antiviral immunity. Sci Immunol. (2022) 7:eabj8760. doi: 10.1126/sciimmunol.abj8760, PMID: 36269840

[23] Galvez NMS Bohmwald K Pacheco GA Andrade CA Carreno LJ Kalergis AM. Type I natural killer T cells as key regulators of the immune response to infectious diseases. Clin Microbiol Rev. (2021) 34:e00232-20. doi: 10.1128/CMR.00232-20, PMID: 33361143 PMC7950362

[24] Wang Z. The intellectual base and research fronts of IL-18: A bibliometric review of the literature from WoSCC (2012-2022). Cell Prolif. (2024) 57:e13684. doi: 10.1111/cpr.13684, PMID: 39188114 PMC11533073

[25] Jin Y Wan K Liu C Cheng W Wang R. Mechanisms of exercise intervention in type 2 diabetes: a bibliometric and visualization analysis based on CiteSpace. Front Endocrinol (Lausanne). (2024) 15:1401342. doi: 10.3389/fendo.2024.1401342, PMID: 39149117 PMC11324446

[26] Benlagha K Kyin T Beavis A Teyton L Bendelac A. A thymic precursor to the NK T cell lineage. Science. (2002) 296:553–5. doi: 10.1126/science.1069017, PMID: 11968185

[27] Zhou D Mattner J Cantu C 3rd Schrantz N Yin N Gao Y. Lysosomal glycosphingolipid recognition by NKT cells. Science. (2004) 306:1786–9. doi: 10.1126/science.1103440, PMID: 15539565

[28] Mattner J Debord KL Ismail N Goff RD Cantu C 3rd Zhou D. Exogenous and endogenous glycolipid antigens activate NKT cells during microbial infections. Nature. (2005) 434:525–9. doi: 10.1038/nature03408, PMID: 15791258

[29] Smyth MJ Crowe NY Pellicci DG Kyparissoudis K Kelly JM Takeda K. Sequential production of interferon-gamma by NK1.1(+) T cells and natural killer cells is essential for the antimetastatic effect of alpha-galactosylceramide. Blood. (2002) 99:1259–66. doi: 10.1182/blood.v99.4.1259, PMID: 11830474

[30] Rahimpour A Koay HF Enders A Clanchy R Eckle SB Meehan B. Identification of phenotypically and functionally heterogeneous mouse mucosal-associated invariant T cells using MR1 tetramers. J Exp Med. (2015) 212:1095–108. doi: 10.1084/jem.20142110, PMID: 26101265 PMC4493408

[31] Andrlova H Miltiadous O Kousa AI Dai A DeWolf S Violante S. MAIT and Vdelta2 unconventional T cells are supported by a diverse intestinal microbiome and correlate with favorable patient outcome after allogeneic HCT. Sci Transl Med. (2022) 14:eabj2829. doi: 10.1126/scitranslmed.abj2829, PMID: 35613281 PMC9893439

[32] Koay HF Gherardin NA Xu C Seneviratna R Zhao Z Chen Z. Diverse MR1-restricted T cells in mice and humans. Nat Commun. (2019) 10:2243. doi: 10.1038/s41467-019-10198-w, PMID: 31113973 PMC6529461

[33] Xu C Obers A Qin M Brandli A Wong J Huang X. Selective regulation of IFN-gamma and IL-4 co-producing unconventional T cells by purinergic signaling. J Exp Med. (2024) 221:e20240354. doi: 10.1084/jem.20240354, PMID: 39560665 PMC11577439

[34] Bendelac A Savage PB Teyton L. The biology of NKT cells. Annu Rev Immunol. (2007) 25:297–336. doi: 10.1146/annurev.immunol.25.022106.141711, PMID: 17150027

[35] Brennan PJ Brigl M Brenner MB. Invariant natural killer T cells: an innate activation scheme linked to diverse effector functions. Nat Rev Immunol. (2013) 13:101–17. doi: 10.1038/nri3369, PMID: 23334244

[36] Kronenberg M Ascui G. The alpha glycolipid rules the NKT cell TCR. J Exp Med. (2025) 222:e20242099. doi: 10.1084/jem.20242099, PMID: 39714312 PMC11665446

[37] Tsuji M Nair MS Masuda K Castagna C Chong Z Darling TL. An immunostimulatory glycolipid that blocks SARS-CoV-2, RSV, and influenza infections. vivo Nat Commun. (2023) 14:3959. doi: 10.1038/s41467-023-39738-1, PMID: 37402814 PMC10319732

[38] Schmieg J Yang G Franck RW Tsuji M. Superior protection against malaria and melanoma metastases by a C-glycoside analogue of the natural killer T cell ligand alpha-Galactosylceramide. J Exp Med. (2003) 198:1631–41. doi: 10.1084/jem.20031192, PMID: 14657217 PMC2194137

[39] Kinjo Y Illarionov P Vela JL Pei B Girardi E Li X. Invariant natural killer T cells recognize glycolipids from pathogenic Gram-positive bacteria. Nat Immunol. (2011) 12:966–74. doi: 10.1038/ni.2096, PMID: 21892173 PMC3178673

[40] Wieland Brown LC Penaranda C Kashyap PC Williams BB Clardy J Kronenberg M. Production of alpha-galactosylceramide by a prominent member of the human gut microbiota. PloS Biol. (2013) 11:e1001610. doi: 10.1371/journal.pbio.1001610, PMID: 23874157 PMC3712910

[41] Cheng TY Praveena T Govindarajan S Almeida CF Pellicci DG Arkins WC. Lipidomic scanning of self-lipids identifies headless antigens for natural killer T cells. Proc Natl Acad Sci U S A. (2024) 121:e2321686121. doi: 10.1073/pnas.2321686121, PMID: 39141352 PMC11348285

[42] Shyanti RK Haque M Singh R Mishra M. Optimizing iNKT-driven immune responses against cancer by modulating CD1d in tumor and antigen presenting cells. Clin Immunol. (2024) 269:110402. doi: 10.1016/j.clim.2024.110402, PMID: 39561929 PMC12145960

[43] Liu X Zhang P Zhang Y Wang Z Xu S Li Y. Glycolipid iGb3 feedback amplifies innate immune responses via CD1d reverse signaling. Cell Res. (2019) 29:42–53. doi: 10.1038/s41422-018-0122-7, PMID: 30514903 PMC6318291

[44] Paget C Deng S Soulard D Priestman DA Speca S von Gerichten J. TLR9-mediated dendritic cell activation uncovers mammalian ganglioside species with specific ceramide backbones that activate invariant natural killer T cells. PloS Biol. (2019) 17:e3000169. doi: 10.1371/journal.pbio.3000169, PMID: 30822302 PMC6420026

[45] Rossjohn J Pellicci DG Patel O Gapin L Godfrey DI. Recognition of CD1d-restricted antigens by natural killer T cells. Nat Rev Immunol. (2012) 12:845–57. doi: 10.1038/nri3328, PMID: 23154222 PMC3740582

[46] Treiner E Lantz O. CD1d- and MR1-restricted invariant T cells: of mice and men. Curr Opin Immunol. (2006) 18:519–26. doi: 10.1016/j.coi.2006.07.001, PMID: 16870416

[47] Melum E Jiang X Baker KD Macedo MF Fritsch J Dowds CM. Control of CD1d-restricted antigen presentation and inflammation by sphingomyelin. Nat Immunol. (2019) 20:1644–55. doi: 10.1038/s41590-019-0504-0, PMID: 31636468 PMC7249499

[48] Rudolph M Wang Y Simolka T Huc-Claustre E Dai L Grotenbreg G. Sortase A-cleavable CD1d identifies sphingomyelins as major class of CD1d-associated lipids. Front Immunol. (2022) 13:897873. doi: 10.3389/fimmu.2022.897873, PMID: 35874748 PMC9301999

[49] Hayashizaki K Kamii Y Kinjo Y. Glycolipid antigen recognition by invariant natural killer T cells and its role in homeostasis and antimicrobial responses. Front Immunol. (2024) 15:1402412. doi: 10.3389/fimmu.2024.1402412, PMID: 38863694 PMC11165115

[50] Mendez Y Vasco AV Ebensen T Schulze K Yousefi M Davari MD. Diversification of a novel alpha-galactosyl ceramide hotspot boosts the adjuvant properties in parenteral and mucosal vaccines. Angew Chem Int Ed Engl. (2024) 63:e202310983. doi: 10.1002/anie.202310983, PMID: 37857582

[51] Speir M Hermans IF Weinkove R. Engaging natural killer T cells as ‘Universal helpers’ for vaccination. Drugs. (2017) 77:1–15. doi: 10.1007/s40265-016-0675-z, PMID: 28005229

[52] Kinjo Y Takatsuka S Kitano N Kawakubo S Abe M Ueno K. Functions of CD1d-restricted invariant natural killer T cells in antimicrobial immunity and potential applications for infection control. Front Immunol. (2018) 9:1266. doi: 10.3389/fimmu.2018.01266, PMID: 29928278 PMC5997780

[53] Cortesi F Delfanti G Casorati G Dellabona P. The pathophysiological relevance of the iNKT cell/mononuclear phagocyte crosstalk in tissues. Front Immunol. (2018) 9:2375. doi: 10.3389/fimmu.2018.02375, PMID: 30369933 PMC6194905

[54] Carnaud C Lee D Donnars O Park SH Beavis A Koezuka Y. Cutting edge: Cross-talk between cells of the innate immune system: NKT cells rapidly activate NK cells. J Immunol. (1999) 163:4647–50. doi: 10.4049/jimmunol.163.9.4647, PMID: 10528160

[55] Zhu T Wang R Miller H Westerberg LS Yang L Guan F. The interaction between iNKT cells and B cells. J Leukoc Biol. (2022) 111:711–23. doi: 10.1002/JLB.6RU0221-095RR, PMID: 34312907

[56] Leadbetter EA Karlsson MCI. Invariant natural killer T cells balance B cell immunity. Immunol Rev. (2021) 299:93–107. doi: 10.1111/imr.12938, PMID: 33438287 PMC8485762

[57] Burn OK Farrand K Pritchard T Draper S Tang CW Mooney AH. Glycolipid-peptide conjugate vaccines elicit CD8(+) T-cell responses and prevent breast cancer metastasis. Clin Transl Immunol. (2022) 11:e1401. doi: 10.1002/cti2.1401, PMID: 35795321 PMC9250805

[58] Grasso C Field CS Tang CW Ferguson PM JC B Anderson RJ. Vaccines adjuvanted with an NKT cell agonist induce effective T-cell responses in models of CNS lymphoma. Immunotherapy. (2020) 12:395–406. doi: 10.2217/imt-2019-0134, PMID: 32316797

[59] Holz LE Chua YC de Menezes MN Anderson RJ Draper SL Compton BJ. Glycolipid-peptide vaccination induces liver-resident memory CD8(+) T cells that protect against rodent malaria. Sci Immunol. (2020) 5:eaaz8035. doi: 10.1126/sciimmunol.aaz8035, PMID: 32591409

[60] Townsend MJ Weinmann AS Matsuda JL Salomon R Farnham PJ Biron CA. T-bet regulates the terminal maturation and homeostasis of NK and Valpha14i NKT cells. Immunity. (2004) 20:477–94. doi: 10.1016/s1074-7613(04)00076-7, PMID: 15084276

[61] Kim PJ Pai SY Brigl M Besra GS Gumperz J Ho IC. GATA-3 regulates the development and function of invariant NKT cells. J Immunol. (2006) 177:6650–9. doi: 10.4049/jimmunol.177.10.6650, PMID: 17082577

[62] Savage AK Constantinides MG Han J Picard D Martin E Li B. The transcription factor PLZF directs the effector program of the NKT cell lineage. Immunity. (2008) 29:391–403. doi: 10.1016/j.immuni.2008.07.011, PMID: 18703361 PMC2613001

[63] LaMarche NM Kane H Kohlgruber AC Dong H Lynch L Brenner MB. Distinct iNKT cell populations use IFNgamma or ER stress-induced IL-10 to control adipose tissue homeostasis. Cell Metab. (2020) 32:243–58 e6. doi: 10.1016/j.cmet.2020.05.017, PMID: 32516575 PMC8234787

[64] Qian G Qin X Zang YQ Ge B Guo TB Wan B. High doses of alpha-galactosylceramide potentiate experimental autoimmune encephalomyelitis by directly enhancing Th17 response. Cell Res. (2010) 20:480–91. doi: 10.1038/cr.2010.6, PMID: 20084083

[65] Raus S Lopez-Scarim J Luthy J Billerbeck E. Hepatic iNKT cells produce type 2 cytokines and restrain antiviral T cells during acute hepacivirus infection. Front Immunol. (2022) 13:953151. doi: 10.3389/fimmu.2022.953151, PMID: 36159876 PMC9501689

[66] Giannou AD Kempski J Shiri AM Lucke J Zhang T Zhao L. Tissue resident iNKT17 cells facilitate cancer cell extravasation in liver metastasis via interleukin-22. Immunity. (2023) 56:125–42 e12. doi: 10.1016/j.immuni.2022.12.014, PMID: 36630911 PMC9839362

[67] Hess NJ SB N Bobeck EA McDougal CE Ma S Sauer JD. iNKT cells coordinate immune pathways to enable engraftment in nonconditioned hosts. Life Sci Alliance. (2021) 4:e202000999. doi: 10.26508/lsa.202000999, PMID: 34112724 PMC8200291

[68] Ghraieb A Keren A Ginzburg A Ullmann Y Schrum AG Paus R. iNKT cells ameliorate human autoimmunity: Lessons from alopecia areata. J Autoimmun. (2018) 91:61–72. doi: 10.1016/j.jaut.2018.04.001, PMID: 29680372

[69] Simonetta F Lohmeyer JK Hirai T Maas-Bauer K Alvarez M Wenokur AS. Allogeneic CAR invariant natural killer T cells exert potent antitumor effects through host CD8 T-cell cross-priming. Clin Cancer Res. (2021) 27:6054–64. doi: 10.1158/1078-0432.CCR-21-1329, PMID: 34376537 PMC8563377

[70] O’Neal J Mavers M Jayasinghe RG DiPersio JF. Traversing the bench to bedside journey for iNKT cell therapies. Front Immunol. (2024) 15:1436968. doi: 10.3389/fimmu.2024.1436968, PMID: 39170618 PMC11335525

[71] Guo J Bao X Liu F Guo J Wu Y Xiong F. Efficacy of invariant natural killer T cell infusion plus transarterial embolization vs transarterial embolization alone for hepatocellular carcinoma patients: A phase 2 randomized clinical trial. J Hepatocell Carcinoma. (2023) 10:1379–88. doi: 10.2147/JHC.S416933, PMID: 37637501 PMC10455792

[72] Rowan AG Ponnusamy K Ren H Taylor GP Cook LBM Karadimitris A. CAR-iNKT cells targeting clonal TCRVbeta chains as a precise strategy to treat T cell lymphoma. Front Immunol. (2023) 14:1118681. doi: 10.3389/fimmu.2023.1118681, PMID: 36936927 PMC10019783

[73] Heczey A Xu X Courtney AN Tian G Barragan GA Guo L. Anti-GD2 CAR-NKT cells in relapsed or refractory neuroblastoma: updated phase 1 trial interim results. Nat Med. (2023) 29:1379–88. doi: 10.1038/s41591-023-02363-y, PMID: 37188782

[74] Exley MA Friedlander P Alatrakchi N Vriend L Yue S Sasada T. Adoptive transfer of invariant NKT cells as immunotherapy for advanced melanoma: A phase I clinical trial. Clin Cancer Res. (2017) 23:3510–19. doi: 10.1158/1078-0432.CCR-16-0600, PMID: 28193627 PMC5511564

[75] Li YR Zhou K Zhu Y Halladay T Yang L. Breaking the mold: Unconventional T cells in cancer therapy. Cancer Cell. (2024) 43:317–322. doi: 10.1016/j.ccell.2024.11.010, PMID: 39672171

[76] Courtney AN Tian G Metelitsa LS. Natural killer T cells and other innate-like T lymphocytes as emerging platforms for allogeneic cancer cell therapy. Blood. (2023) 141:869–76. doi: 10.1182/blood.2022016201, PMID: 36347021 PMC10023720

[77] Lee MS Webb TJ. Novel lipid antigens for NKT cells in cancer. Front Immunol. (2023) 14:1173375. doi: 10.3389/fimmu.2023.1173375, PMID: 37908366 PMC10613688

[78] Lameris R Shahine A Pellicci DG Uldrich AP Gras S Le Nours J. A single-domain bispecific antibody targeting CD1d and the NKT T-cell receptor induces a potent antitumor response. Nat Cancer. (2020) 1:1054–65. doi: 10.1038/s43018-020-00111-6, PMID: 35122066

[79] Kuen DS Hong J Lee S Koh CH Kwak M Kim BS. A personalized cancer vaccine that induces synergistic innate and adaptive immune responses. Adv Mater. (2023) 35:e2303080. doi: 10.1002/adma.202303080, PMID: 37249019

[80] Pandey P Kim SH Subedi L Mujahid K Kim Y Cho YC. Oral lymphatic delivery of alpha-galactosylceramide and ovalbumin evokes anti-cancer immunization. J Control Release. (2023) 356:507–24. doi: 10.1016/j.jconrel.2023.03.010, PMID: 36907564

[81] Wei P Romano C Li C Clergeaud G Andresen TL Henriksen JR. An intranasal cationic liposomal polysaccharide vaccine elicits humoral immune responses against Streptococcus pneumoniae. Commun Biol. (2024) 7:1158. doi: 10.1038/s42003-024-06806-1, PMID: 39284859 PMC11405767

[82] Shute T Amiel E Alam N Yates JL Mohrs K Dudley E. Glycolipid-Containing Nanoparticle Vaccine Engages Invariant NKT Cells to Enhance Humoral Protection against Systemic Bacterial Infection but Abrogates T-Independent Vaccine Responses. J Immunol. (2021) 206:1806–16. doi: 10.4049/jimmunol.2001283, PMID: 33811104

[83] Li K Hu X Tu XY Xian MY Huang LL Huang T. Enhancing COVID-19 vaccine efficacy: dual adjuvant strategies with TLR7/8 agonists and glycolipids. J Med Chem. (2024) 67:21916–33. doi: 10.1021/acs.jmedchem.4c01801, PMID: 39648985

[84] Hu X Xian MY Wang XF Zou GQ Luo R Peng H. Conformationally restricted analogues of alpha-galactosylceramide as adjuvant in COVID-19 subunit vaccine. ACS Med Chem Lett. (2023) 14:1647–55. doi: 10.1021/acsmedchemlett.3c00154, PMID: 38116441 PMC10726466

[85] Li X Huang J Kaneko I Zhang M Iwanaga S Yuda M. A potent adjuvant effect of a CD1d-binding NKT cell ligand in human immune system mice. Expert Rev Vaccines. (2017) 16:73–80. doi: 10.1080/14760584.2017.1256208, PMID: 27801602 PMC5526659

[86] Varghese B Lynch L Vriend LE Draganov D Clark JM Kissick HT. Invariant NKT cell-augmented GM-CSF-secreting tumor vaccine is effective in advanced prostate cancer model. Cancer Immunol Immunother. (2022) 71:2943–55. doi: 10.1007/s00262-022-03210-8, PMID: 35523889 PMC10992623

[87] Yang D Li X Li J Liu Z Li T Liao P. Fully Synthetic TF-Based Self-Adjuvanting Vaccine Simultaneously Triggers iNKT Cells and Mincle and Protects Mice against Tumor Development. J Med Chem. (2024) 67:17640–56. doi: 10.1021/acs.jmedchem.4c01631, PMID: 39302195

[88] Kamata T Suzuki A Mise N Ihara F Takami M Makita Y. Blockade of programmed death-1/programmed death ligand pathway enhances the antitumor immunity of human invariant natural killer T cells. Cancer Immunol Immunother. (2016) 65:1477–89. doi: 10.1007/s00262-016-1901-y, PMID: 27631416 PMC5099366

[89] Sharma AK LaPar DJ Stone ML Zhao Y Mehta CK Kron IL. NOX2 activation of natural killer T cells is blocked by the adenosine A2A receptor to inhibit lung ischemia-reperfusion injury. Am J Respir Crit Care Med. (2016) 193:988–99. doi: 10.1164/rccm.201506-1253OC, PMID: 26757359 PMC4872653

[90] Liu D Song L Wei J Courtney AN Gao X Marinova E. IL-15 protects NKT cells from inhibition by tumor-associated macrophages and enhances antimetastatic activity. J Clin Invest. (2012) 122:2221–33. doi: 10.1172/JCI59535, PMID: 22565311 PMC3366399

[91] Tian C Wang Y Su M Huang Y Zhang Y Dou J. Motility and tumor infiltration are key aspects of invariant natural killer T cell anti-tumor function. Nat Commun. (2024) 15:1213. doi: 10.1038/s41467-024-45208-z, PMID: 38332012 PMC10853287

[92] Zhang H Chen S Zhang Y Tian C Pan J Wang Y. Antigen Priming Induces Functional Reprogramming in iNKT Cells via Metabolic and Epigenetic Regulation: An Insight into iNKT Cell-Based Antitumor Immunotherapy. Cancer Immunol Res. (2023) 11:1598–610. doi: 10.1158/2326-6066.CIR-23-0448, PMID: 37756568

[93] Fu S He K Tian C Sun H Zhu C Bai S. Impaired lipid biosynthesis hinders anti-tumor efficacy of intratumoral iNKT cells. Nat Commun. (2020) 11:438. doi: 10.1038/s41467-020-14332-x, PMID: 31974378 PMC6978340

[94] Das R Guan P Wiener SJ Patel NP Gohl TG Evans E. Enhancing the antitumor functions of invariant natural killer T cells using a soluble CD1d-CD19 fusion protein. Blood Adv. (2019) 3:813–24. doi: 10.1182/bloodadvances.2018028886, PMID: 30858151 PMC6418505

[95] Htwe KSS Soontrapa K Prasopporn S Chusorn P Okada S Jirawatnotai S. Vorinostat restores iNKT cell functionality in aggressive cholangiocarcinoma. BioMed Pharmacother. (2025) 186:117964. doi: 10.1016/j.biopha.2025.117964, PMID: 40101585

